# Limited awareness prevents the use of compost and biochar as soil amendments by Ghanaian cocoa farmers

**DOI:** 10.1007/s44279-026-00754-6

**Published:** 2026-08-31

**Authors:** Dadson Awunyo-Vitor, Andrew Daymond, Amos Quaye, Godfred Kweku Awudzi, Laura Atuah, Lumbani Mwafulirwa, John Hammond, Chris Turnbull, Fiona Lahive, Sean Coole, Steve Robinson, Paul Hadley, Tom Sizmur

**Affiliations:** 1 Department of Agricultural Economics, Agribusiness and Extension, Kwame Nkrumah University of Science and Technology, Kumasi, Ghana; 2 School of Agriculture, Policy and Development, University of Reading, Reading, UK; 3 Cocoa Research Institute of Ghana, Tafo, Ghana; 4 Department of Horticulture, Kwame Nkrumah University of Science and Technology, Kumasi, Ghana; 5 Department of Geography and Environmental Science, University of Reading, Reading, UK

**Keywords:** Soil amendments, Compost, Biochar, Cocoa, Farmer, Questionnaire

## Abstract

Organic soil amendments can be used to reduce the environmental impact of cocoa farming, increase sustainability, and protect livelihoods. This study aimed to explore Ghanaian cocoa farmers’ awareness and usage of compost and biochar on their farms and to make recommendations to increase adoption. A survey was conducted with 150 cocoa farmers, randomly drawn from a population of 245 across five cocoa-growing districts and representing four different soil types across the five major agroecological regions where cocoa is grown in Ghana. The survey was followed by a focus group discussion to better understand responses to the survey questions. Farmers attributed low cocoa yields to declining soil fertility and the high cost of farm inputs, particularly chemical fertilisers. Although some farmers were aware of compost use in vegetable production, none of the farmers surveyed used compost or biochar on their cocoa farms. The findings suggest that the reason for the lack of adoption is limited awareness. However, farmers were willing to learn how to prepare compost and biochar or to buy these amendments. Therefore, embedding training on the preparation and use of compost and biochar into equitable and inclusive programmes led by the Ghanaian Cocoa Board (COCOBOD), licensed buying companies (LBCs), certification schemes (e.g. Rainforest Alliance, Fairtrade, Organic), and farmer associations (cooperatives) is key to increasing adoption, elevating sustainability, and improving livelihoods. These organisations could also collectively support and subsidise the production and application of organic amendments by enterprises as a service provided to farmers.

**Supplementary Information:**

The online version contains supplementary material available at https://doi.org/10.1007/s44279-026-00754-6.

## Introduction

About 71% of the world’s cocoa is grown in West Africa, with Ghana being the second largest producer after Côte d’Ivoire [1]. In Ghana, an estimated 800,000 farmers cultivate cocoa [2] on relatively small plots of land, typically less than 5 ha [3], thus making it an important source of rural livelihoods. Despite the emergence of the oil sector, agriculture still contributes around 22% to Ghana’s GDP, provides employment to about 45% of the population, and contributes about 45% of all export earnings [4]. Cocoa is therefore very important to the Ghanaian economy due to its contribution to the agricultural sector and export earnings. Cocoa supported the overall growth in the Ghanaian agricultural sector by about 28% [4], and is the third biggest export commodity and foreign exchange earner, thereby contributing considerably to the nation’s economic development.

Due to the importance of the cocoa sector, the government of Ghana has introduced several measures to improve cocoa production. These measures include: support for the rehabilitation programme, fertiliser credit systems, and the liberalization of internal marketing of cocoa [5]. In addition, the government has encouraged the formation of farmer associations and cooperatives, which are used as a vehicle to educate and train farmers on cocoa production practices. Nevertheless, productivity per hectare has not seen much improvement over the last decade. It appears that historic increases in Ghana’s cocoa output levels have been primarily a result of the increase in area under cocoa rather than yield increases, and this trend is also observed in other West African cocoa producing countries [6].

Yields between cocoa farms are highly variable (ranging ~ 100 to > 1000 kg ha^-1^) and agronomic management appears to be a major determinant of such variation [7]. Depletion of soil organic matter and reduction in soil fertility as a result of continuous cropping, coupled with inappropriate and inadequate applications of soil amendments, is a major cause of low productivity in cocoa systems across West Africa [8]. Amponsah-Doku et al., [6] identified that improvements to soil fertility (including the use of compost) are important to closing the gap between potential and realized yield. In addition to loss of soil fertility, poor farm management practices and aging cocoa trees can also contribute to low productivity [5]. There is a need to improve cocoa yields in order to make smallholder farming a sustainable livelihood, attract the unemployed youth to cocoa farming, and curb rural to urban migration [9, 10]. Cost effective soil fertility management, that re-supplies all needed nutrients to soils is essential for high cocoa productivity and is critical for the economic viability of smallholder farming enterprises [11].

Soil amendments are any material that has been added to the soil to improve its chemical or physical properties [12]. The two major classes of soil amendments are organic and inorganic. Inorganic soil amendments include chemical fertilisers used by the farmers to improve productivity on their farms. The government of Ghana, in an attempt to improve cocoa productivity, supported cocoa farmers with free chemical fertilisers through the Ghana Cocoa Board (COCOBOD). However, in most cases the amount supplied to farmers is inadequate, and the use of these fertilisers can decouple nitrogen and phosphorous cycles in agricultural ecosystems [13]. Nevertheless, farmers have adopted inorganic soil amendments. Inorganic fertilisers have the potential to make the soil acidic, which reduces the availability and uptake of some nutrients [14], with negative consequences on crop yields. Furthermore, other harmful effects of inorganic fertilisers include pollution of surface water bodies and air pollution. Due to the price of these inorganic soil amendments, most cocoa farmers are unable to afford the appropriate quantity (in addition to those supplied by the government) needed for optimum productivity [15].

Organic soil amendments such as compost and biochar have a positive impact on soils because they provide plant nutrients, build soil organic matter, improve soil structure, and increase microbial activity [16]. Organic soil amendments are particularly effective at increasing crop yields through improved soil fertility [17]. Compost may substitute for mineral fertiliser, which is expensive and can acidify soils over time [18]. Compost has the potential to improve the soil quality and soil organic matter content as well as prevent loss of soil nutrients through processes such as nitrogen via volatilization [19]. Compost has also been found to suppress the population of nematodes in cocoa farms and increase soil health [20]. Biochar s made by burning organic material in the absence of oxygen through a process known as pyrolysis. The resulting biochar is a recalcitrant source of carbon, improves soil structure, increases water holding capacity, and alleviates soil acidity; leading to improved crop productivity [21, 22]. The application of compost and biochar to degraded soils can improve soil physical and chemical characteristics and support crop growth.

**Fig. 1:**
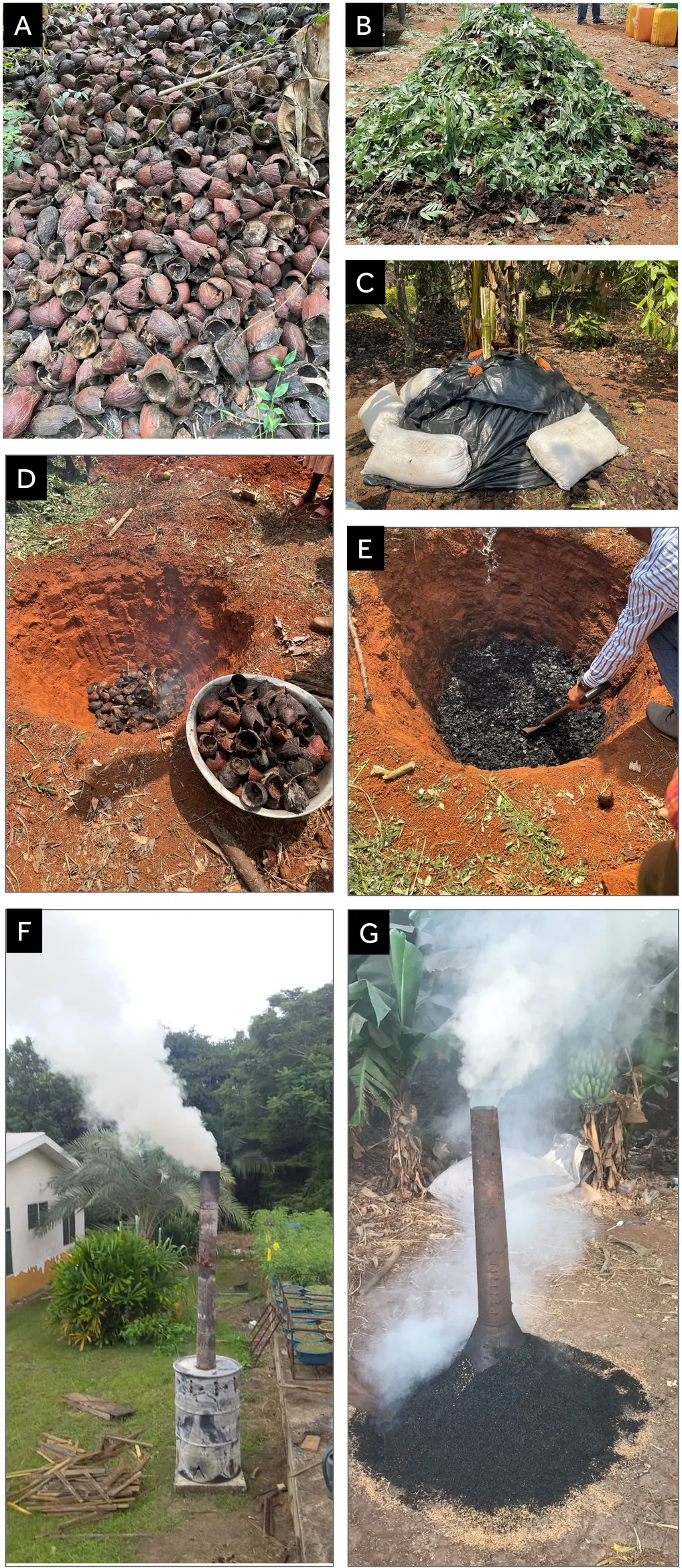
Images of on-farm compost and biochar preparation from cocoa pod husks (**A**). Husks are composted by mixing with the trimmings of leguminous trees (**B**) and covered with a ventilated plastic sheet (**C**). Husks can be pyrolysed using the open pit method (**D** and **E**), whereas woodchips and corn cobs can by pyrolysed using the ELSA barrel (**F**), and rice husks or sawdust pyrolysed using the Japanese Retort Stove (**G**)

Both compost and biochar can be prepared from a range of organic wastes using a variety of methods that are accessible to farmers who have received training (Fig. 1). In particular, both can be produced from on-farm waste such as cocoa pod husks [23], which are in abundance on cocoa farms, and thus can be used to improve soil fertility and cocoa productivity [24]. Cocoa pod husks contain a large amount of phosphorous and potassium [25]. However, many farmers consider cocoa pod husks as a waste, and a vast quantity is disposed of after beans have been extracted. It therefore appears that farmers are unaware of its efficacy as an organic fertiliser source. Some farmers use cocoa pod husks in the manufacturing of local black soap, but the majority are seemingly oblivious of the ameliorating effects of cocoa pod husk-derived soil amendments on nutrient-depleted soils.

Although the material to produce soil amendments is readily available, and can be easily prepared, the unanswered question is why cocoa farmers are not using compost and biochar to improve soil fertility on their farms. This study was designed to fill this knowledge gap by quantifying the awareness and usage of compost and biochar by cocoa farmers in Ghana. While several prior studies have explored the adoption and benefits of ‘supply side interventions’, such as seedling provision, pruning services, pesticides, and inorganic fertilisers [26–29], our survey is novel because we focus on the adoption of practices (compost and biochar) which farmers could make and apply themselves without requiring external inputs or services. The findings of the survey lead to recommendations on how adoption can be encouraged and increased within Ghana and further afield.

## Methods

This study was undertaken in the southern part of Ghana where cocoa production takes place. A multi-stage selection technique was adopted to select the respondents. In the first stage a soil map was used to identify four soil types across the five major agroecological regions where cocoa is grown in Ghana (Wet Evergreen, Moist Evergreen, Moist Deciduous Evergreen, Moist Deciduous South-East Ecotype, and Dry Semi-Deciduous), following [30]. These soil types were Ferralsols, Lixisols, Nitosols and Acrisols. During the second stage, five cocoa districts (Aiyinase, Assin Foso, Dadiesoaba, Kade, and Nyarkrom) were randomly selected to ensure representation of each of these soil types because the use of soil amendments may differ in efficacy between soil types. During the third stage, three operational areas (i.e. towns) were randomly sampled from the selected cocoa districts where questionnaire respondents were recruited. The questionnaire was undertaken at a public/community location prior to farmers from each district participating in compost and biochar making training events and hosting on-farm field trials in each district as part of our wider project. Cocoa Extension Agents from the Cocoa Health and Extension Division (CHED) of COCOBOD were approached to prepare a list of cocoa farmers they were working with in each operational area. Within all areas combined, a population of 245 farmers were identified as willing to take part in the study. We used Table 1 in Krejcie and Morgan [31] to identify that a sample size of 150 farmers would adequately represent the 245 farmers in our population. We therefore emphasise that our survey is representative of the farmers already working with Cocoa Extension Agents within the operational areas that we undertook the survey rather than all cocoa farmers in Ghana. However, we have attempted to ensure that these areas are as representative as possible of the wider cocoa growing regions. A simple random sampling technique was then used to select 150 individual respondents from the 245 candidate farmers. We acknowledge that our participants are unlikely to represent farmers within the operational areas who are not in contact with the Cocoa Extension Agents.

A structured cross-sectional questionnaire was created and used to collect data and information from each respondent cocoa farmer. The questions asked were divided into sections which included demographic characteristics of the farmer, information about their cocoa farm, the management practices they carry out on their farm, their knowledge of soil fertility, their awareness and usage of organic and inorganic fertilisers, their use of the cocoa pod husks, and then specifically their awareness of, use of, and willingness to use both compost and biochar. The farmers were asked to state the challenges they faced with respect to cocoa farming and then rate or rank these challenges from the most important challenge that needed immediate attention to less critical challenges. The full questionnaire is included as Supplementary material.

Following the collection and analysis of the questionnaire, a focus group discussion was initiated to help better understand the farmers responses to some specific questions. Particularly where the responses to the questions were different to our expectations. The questions asked in the focus group are provided in the Supplementary material. The focus group comprised of 8 participants. All participants were selected by Cocoa Extension Agents using a purposive sampling approach based on their position in the communities, leading to a high level of knowledge and insight about the issues raised in the focus group. The 8 focus group participants included 2 male farmers with more than 10 years of cocoa farming experience, 2 female farmers with more than 10 years of cocoa farming experience, 2 farmers with at least high school level education, and 2 cocoa cooperative chair persons. Insights emerging from the focus group were used to interpret the responses that farmers gave to questions, based on consensus among the participants.

The identity of both survey and focus group participants was kept confidential and the raw data was kept on a password-protected computer. Data and information collected from the respondents were analysed using descriptive statistics and content analysis. The socioeconomic characteristics of the respondents were presented using descriptive statistics such as frequency tables and percentages. The Kendall’s Coefficient of Concordance (W) was computed in Excel to assess agreement among the farmers with respect to the rating or ranking of challenges. The method by which W was calculated is presented in the Supplementary materials.

## Results and discussion

### Demographic characteristics of the respondents

The demographic characteristics of the farmers surveyed are summarised in Tables 1 and 2 (categorical variables in Table 1 and continuous variables in Table 2). The results revealed that 84% of the respondents were household heads. In all, 113 male and 37 female farmers were interviewed representing 75.3% and 24.7% of respondents, respectively. This representation aligns with the results of a similar survey published in 2017, which revealed that 74.8% of the respondents were male while 25.2% were female [32]. Male dominance of the cocoa sector was similarly reported by Kongor et al., [5] and was attributed to the inheritance of land and other assets by males to a greater extent than females. It has been reported previously that female cocoa farmers are less technically efficient than male cocoa farmers and this may be due to differences in resources, education, farm size, and the requirement for female farmers to engage in non-farming activities [33]. These differences may contribute to a gender yield gap that is often observed on smallholder farms [34] and can result in lower income of female farmers, compared to their male counterparts [35]. These gendered differences may result in a different capacity for male farmers and female farmers to adopt the use of organic soil amendments.

The majority (58.7%) of the respondents reported that they were indigenous, while 41.3% self-identified as immigrants (Table 1). This result is similar to the survey by Cocoa Alliance, which found 58.9% of the respondents to be natives [32]. In a focus group discussion, it was revealed that respondents who self-identified as immigrants were all second or third generation immigrants. They were the children and grandchildren of those who originally established the cocoa farms. Further analysis revealed that over 90% of the respondents self-identifying as migrants were from other regions in Ghana while the rest were from other West African countries. Farmers that are natives from the community where their farms are located are more likely to have extended family members living nearby. As a result, farmers that are natives from the community where their farms are located are more likely to receive support in times of need from their extended family members. Family labour is considered a household asset and the availability of family labour is positively associated with the adoption of inorganic fertilisers as households can reduce labour cost and hence spend more on fertiliser application [36].

The majority of the respondents (82.7%) were married, 15% were divorced, and 4% were single (Table 1). This result is not surprising because most of the farms belonging to questionnaire respondents are located near villages and these villages are generally at quite a distance from the nearest commercial town. Hence, married farmers are better placed to balance travel and farm labour under these conditions than unmarried farmers. Duncan [37] explains that women who support husbands with farm labour on Ghanaian cocoa farms expect to receive land or cocoa farms by way of reciprocity for their labour. However, Dalaa et al., [38] state that husbands are generally not obligated to provide farm labour to their wives, which results in an unequal labour exchange and ultimately means that female cocoa farmers in Ghana have less assured access to farm labour than male cocoa farmers.

The majority of the farmers had some formal education. Only 20.7% did not have any formal education. In all, 9.3% had only primary education, and close to half of the respondents (42%) had middle school and/or junior high school education. There were five respondents with vocational training and three respondents had tertiary education (Table 1). A low level of education has previously been highlighted as a barrier to the adoption of new cocoa production technologies recommended by the Cocoa Research Institute of Ghana [27] but education and experience were more recently observed to be positively associated with the adoption of organic fertilisers by Ghanaian cocoa farmers [39]. All the farmers interviewed stated that they were able to apply pesticides and inorganic fertilisers using the recommended dose. Therefore, the fairly high level of education implies that the majority of farmers are capable of learning and adopting new agricultural technologies, such as compost and biochar.

**Table 1:** Demographic characteristic of the respondents–categorical variables

Questions/statements	Sub-Levels	Frequency	Percentage (%)
Are you the head of the household?	Yes	126	84.0
	No	24	16.0
Gender of respondents	Male	113	75.3
	Female	37	24.7
Are you a native?	Yes	88	58.7
	No	62	41.3
Marital status of the respondents	Married	124	82.7
	Cohabitating	1	0.7
	Separated	1	0.7
	Widowed	5	3.3
	Divorced	15	10.0
	Single	4	2.4
Educational level of the respondents	No formal	31	20.7
	Primary	14	9.3
	JHS/Middle school	63	42.0
	SHS/Secondary school	31	20.7
	Vocational/Training colleges	5	3.3
	Tertiary /University	3	2.0
	Others/ Professional qualification	3	2.0
Do you access income from other sources apart from Cocoa farming?	Yes	113	75.3
	No	37	24.7

About 75% of the farmers interviewed had additional sources of income (Table 1). Most of the farmers (93.8%) derived their additional income from self-employment while the rest (6.2%) did so from being employees. A multiple response analysis revealed that about 72% of those in self-employment are petty traders, 13% are artisans, and 2.5% work as farm labourers on other farms. The farmers can use income from other sources to support their cocoa farming activities when the need arises. However, the proportion of their income from additional sources is low for most of the respondents. It has been found that off-farm income and annual income from cocoa both positively influence the likelihood of Ghana’s cocoa farmers to adopt climate change adaptation technology [40]. Because most of the farmers derived over 70% of their annual income from cocoa cultivation, they are more likely to be motivated to invest in cocoa production by adopting innovations that will increase yield and income, but they are also likely to be less resilient to climate shocks [41].

It was revealed in a focus group discussion that most of the farmers are engaged in other income generating activities because they cannot increase their income by expanding the area of cocoa under cultivation due to the lack of available land. Moreover, yields and, consequently, income from cocoa farming tends to fluctuate significantly. Therefore, farmers engage in other income generating activities to supplement their income from cocoa. One of the farmers stated, “*in fact the money from cocoa is inadequate for me and my family to survive for a year*”. The COCOBOD, non-governmental organisations (NGOs), and chocolate manufacturers encourage cocoa farmers to engage in alterative livelihood activities to supplement their cocoa farming income by providing training in entrepreneurship for the cocoa farmers [42].

The minimum age of the respondents was 24 years with the maximum age being 86, with an average age of about 51 years (Table 2). This age profile is similar to the results of a survey conducted by Asamoah and Owusu-Ansah [32], which found the average age of respondents to be about 49 years. The average age of 51 years implies a need to encourage young people to venture into cocoa farming. This is very important in the face of high unemployment levels. However, the reasons for reluctance to engage in cocoa farming by young people is complex and includes a perceived lack of status and prestige in farming and the lack of infrastructure and amenities in rural communities [9]. It is therefore unlikely that greater yields or on-farm incomes alone will encourage young Ghanaians to engage in cocoa farming as a source of employment. The question “why are the youth not interested in farming?” was posed during the focus group discussion to understand the lack of interest of the youth in cocoa farming. One of the farmers stated, *“Some of the youth have a lifestyle which is fuelled by illegal mining popularly referred to as “galamsey” hence they are not interested in farming*”. The sale of cocoa farms to illegal miners is driven by short term financial pressures, but then has a subsequent impact cocoa yields on neighbouring and downstream farms, thus compounding the problem [43]. Another farmer stated, “*Some of the youth think farming is punishment not business. They think it’s better to engage in illegal mining than farming*”. The focus group discussion also revealed that some of the youth are willing to venture into cocoa farming, but they do not have access to land, which one focus group participant attributed to the “*population growth and alterative use of land*,* including illegal mining”.*

**Table 2:** Demographic characteristics of the respondentscontinuous variables

Demographic characteristics	Minimum	Maximum	Mean	Std. Deviation
Age of the respondents	24.00	86.00	50.90	12.55
Experience in cocoa farming	1.00	43.00	16.56	8.80
Number of years stayed in the community	2.00	82.00	36.30	18.62
Educational level (years)	0.00	20.00	7.58	4.80
Household size	1.00	21.00	6.91	4.36
Number of plots of cocoa farms	1.00	3.00	1.93	2.07
Size of Cocoa farm (Ha)	0.2	10.00	2.14	4.04

The number of years of experience held by the farmers in cocoa cultivation ranged between 1 and 43 years, with an average of 16 years (Table 2). Since most of the farmers have several years of experience in cocoa cultivation, they are in a position to integrate useful innovations that can improve their farming practices. Avane et al., [39] found that the number of years of experience undertaking cocoa farming decreased a cocoa farmer’s likelihood of adopting organic fertiliser because they are more likely to rely on their experience instead of adopting new practices. However, farmers who had contacts with extension agents were more likely to adopt organic fertilisers because they believed their advice to be impartial and not motivated by profit. Appropriate training resources are therefore very important to ensure the introduction of new technologies into the cocoa sector [44].

### Soil fertility and fertiliser use

The majority (92%) of the farmers interviewed indicated that they could identify poor soil (Table 3). These farmers were then asked an open question about how they identify poor soil. The way the respondents answered this question reflects the fact that most of the farmers were members of the Cocoa Abrabopa Association (CAA); an association which trains farmers to practice soil fertility management [45]. Some of the methods farmers use to identify poor soils include recognising unhealthy leaves on the cocoa tree, the number of pods that wilted before maturity, the size of beans in the pod, and total farm output or yield over time. These responses closely match the methods that CAA members are trained to use to identify poor soil.

With respect to changes in soil fertility, about 67% of the farmers reported a decline in soil fertility over the years and yields were also low and declining (Table 3). Amponsah-Doku et al., [6] note that in some cases, poor soil fertility may be linked to increasing soil acidity due to the application of acid-forming fertilisers, which impedes the availability of nutrients. The perceived decline in soil fertility is supported by Essougong et al., [46] in a study that also revealed a perceived decline in soil fertility in cocoa farming areas. A survey of soils in Ghanaian cocoa districts reveals low soil organic matter and low pH consistent with a ‘boom-bust cycle’ of cocoa cultivation that mines the soil of nutrients [30]. A minority (18.2%) of the respondents noted that soil fertility had not changed, based on the growth rate of their young cocoa plantations and yield, over time, while 12.7% stated that soil fertility had improved due to appropriate agronomic practices and application of fertiliser resulting in increased yield. This observation supports the long-held knowledge that improved agronomic practices can reduce the yield gap of Ghanaian cocoa farms [47].

**Table 3:** Farmers perception of soil fertility and use of soil amendments

Statement/questions on soil fertility and use of organic fertiliser	Sub-level response	Frequency	Percentage
Could you identify poor soil?	Yes	138	92.0
	No	12	8.0
During the time that you have been cultivating cocoa, have you perceived any changes in soil fertility?	Same	28	18.7
	Decline	101	67.3
	Improved	19	12.7
	Cannot tell	2	1.3
Have you taken action by purchasing fertiliser from input dealers to improve soil fertility on your cocoa farm apart from using fertiliser supplied by COCOBOD?	Yes	123	82.0
	No	27	18.0
Have you ever used organic soil amendments?	Yes	9	6
	No	141	94

All the farmers surveyed in the study used inorganic fertiliser supplied by the COCOBOD for free through the cocoa cooperatives whenever they had access to it. Some farmers complained during the focus group discussion about the continued use of inorganic fertiliser. For example, a farmer stated that the “*fertiliser that they have been using over the years makes the soil very hard when the rains did not come early or are inadequate*,* which affect our cocoa productivity negatively”*. In a focus group discussion, it was revealed that farmers with young, recently established farms were less likely to take action on their own to purchase fertiliser from the input dealers to improve soil fertility unless they had access to free fertiliser via COCOBOD. It is likely that these farms are still benefiting from the flush of nutrients entering the soil after ‘slash and burn’ land clearance during the cocoa farm establishment [6]. However, 82% of thefarmers indicated that, in addition to the fertiliser supplied for free by the COCOBOD, they purchase fertiliser for use on their cocoa farms (Table 3). Farmers indicated that the reason they needed to purchase fertilisers and pesticides was because those supplied by COCOBOD were inadequate and were supplied late.

With respect to the use of organic soil amendments, only a few farmers (6%) indicated that they had ever used these (Table 3). During a focus group discussion, it was revealed that the organic soil amendment used by some farmers was poultry manure. Poultry manure can also be used as a nitrogen source for co-composting with cocoa pod husk residues to produce a soil amendment suitable for crop production [48]. The use of poultry manure as an organic fertiliser has been increasing since the early 2000s in Côte d’Ivoire as part of a ‘frugal innovation’ in response to rising inorganic fertiliser prices, including the use of manure imported from chicken farms in Ghana [49]. Some of the farmers mentioned during the focus groups that about 5 years ago a company came to register cocoa farmers and supplied them with poultry manure at a cost, which they applied to their cocoa trees via broadcasting. Some of the participants stated that due to the odour they decided not to use it, as also reported by Avane et al., [39]. All those who applied the poultry manure agreed that it improved the flowering and fruiting of their trees. One of the farmers stated, “*initially I did not like the odour and the look of the poultry manure but when I applied it to the cocoa tree the yield was good and better than the inorganic fertiliser”.* The farmers attributed their inability to use organic fertiliser to lack of knowledge on the benefit and unavailability of the organic fertiliser, as it is not available for sale on the market. One of the farmers said, “*There is no market where one can purchase organic fertiliser in Ghana”*. This is due to competition for poultry manure increasing recently as vegetable farmers rely on poultry manure to produce organic vegetables [50]. Demand for organic fertiliser in Ghana is currently estimated to be approximately 0.7 million tonnes per year, but has the potential to increase to approximately 2.7 million tonnes per year, highlighting a considerable opportunity for entrepreneurs to establish commercial composting operations [51].

### Cocoa production and farm characteristics

More than half (52.0%) of the land holdings for cocoa cultivation was by sharecropping, followed by inheritance and freeholds (40.7%), while lease and rental arrangements accounted for only 4.7% and 2.7% of the interviewees, respectively (Table 4). The sharecropping arrangement is a cheaper means of accessing land for cocoa farming as there is no cash flow involved compared with the lease and rental arrangement. Generally, there are two main sharecropping land tenure systems operating in the cocoa growing areas in Ghana. These are referred to as ‘*Abunu’* and ‘*Abusa*’. Under the ‘*Abunu*’ land tenure system, the immigrant farmer cultivates the land and upon maturity of the crops, the farm is shared between the landlord and the farmer into two equal parts. In the case of ‘*Abusa*’ land tenure system, the farm is divided into three equal parts and the landlord takes two parts and the tenant takes one part. In these land tenure systems, the farm (including the land) then belongs to the tenants and they can pass it on it to their heirs as long as the cocoa trees remain on the land. It is generally found that women and young farmers generally cultivate smaller cocoa farms which are more likely to be inherited or acquired through sharecropping [38]. However, in the focus group discussion, it was revealed that landowners are now moving away from sharecropping in favour of leasing and renting. This is because the land available for cocoa cultivation keeps dwindling as the population increases and as alternative, more lucrative, uses of land are found. The shifting dynamics of land tenure seems to benefit landowners and new migrants over existing farmers (disproportionately impacting women and young farmers) and may result in a reluctance to rehabilitate old cocoa farms and greater encroachment into forest reserves [52].

Almost half (45.3%) of the farms were aged between 0 and 7 years, 24% fall between 8 and 15 years, 16% were 16 to 30 years old, and 7.3% of the farms were older than 30 years. Thus, two thirds of the farms were less than 15 years old and may require practices that maintain soil fertility rather than practices to rehabilitate soil fertility. Nalley et al., [53] found a phased replacement rate of 5–7% of trees from 5 to 9 years after planting to be more optimal for maximising profits than the current strategy of complete replanting after ~ 26 years. However, the decision about whether to rehabilitate or replace cocoa farms is often driven by the need for a regular flow of products for sale and insufficient attention is given to practices that ensure the capacity of the soil to sustain another cropping cycle [54]. All the farmers interviewed also cultivated food crops, which included cassava, yam, and cocoyam. However, it is known that women farmers cultivate fewer food crops than male farmers because they have smaller farms [38] and are thus more vulnerable to declines in productivity. Eleven of the respondents, constituting 7.3%, indicated that they have abandoned their farms (Table 4). In a focus group discussion, the farmers raised various reasons why they abandoned their farms. The reasons included low productivity, leading to inadequate returns and improper management of the farm. Other reasons given included elderly farmers being unable to manage farms and the unwillingness of family members to take over in their absence or demise. The respondents also indicated that litigation leads to abandonment of farms, but this is usually for a short period.

**Table 4:** Farmer’s cocoa production systems

Questions/statements	Sub-level	Frequency	Percentage
Sampled farmers system of land holding	Inherited/freehold	61	40.7
	Lease	7	4.7
	Rent	4	2.7
	Sharecropping	78	52.0
Sharecropping type engaged in by the respondent farmers	Abunu	63	80.8
	Abusa	15	19.2
Age of Cocoa farms of the farmers interviewed	0–7 years	68	45.3
	8–15 years	36	24.0
	16–30 years	24	16.0
	Over 30 years	11	7.3
	Abandoned	11	7.3
Cocoa Varieties planted by the farmers (multiple responses)	Amelonado	21	13.60
	Hybrid	92	59.7
	Both Hybrid & Amelonado	41	26.6
Are you a members of Farmer Based Organisation (cocoa cooperatives)?	Yes	142	94.7
	No	8	5.3
Do you have regular visit by Cocoa Extension Agents?	Yes	140	93.3
	No	10	6.7

The majority (87%) of the interviewed farmers had two plots upon which they cultivate cocoa. A few (13.3%) of the farmers cultivated traditional Amelonado only, while the majority (60%) of the farmers interviewed cultivated Hybrid cocoa only (mixtures of progenies of bi-parental crosses produced by the COCOBOD Seed Production Division), and the rest (26.7%) cultivated a mixture (Table 4). The high level of adoption of the Hybrid varieties is unsurprising given that two thirds of the farms were less than 15 years old. It is also known that younger farmers and farmers with a higher education status are more likely to adopt new technologies, such as the newer Hybrid varieties, since they are more capable of receiving and digesting information disseminated by extension agencies through the media [27].

The majority (94.7%) of the respondents were members of farmer-based organizations (i.e. cooperatives and farmer associations). Cocoa cooperatives are an initiative introduced by the COCOBOD to enable them to provide input and service as well as training to cocoa farmers, but the partial liberalisation of the Ghanaian cocoa market has delegated responsibility for cocoa procurement and some of the associated extension services to Licensed Buying Companies, who encourage and facilitate cooperatives and farmer associations [55]. Cooperatives make it easier for farmers to access training, credit, and participation in certification schemes (e.g. Rainforest Alliance, Fairtrade, Organic), which ultimately increase the sustainability of cocoa farms [56]. The majority of the participants in the focus group discussions alluded to the fact that the co-operatives have helped them to access inputs and training. Respondents reported that, before the cooperatives were formed, it was difficult for them to access farm inputs and machinery to prune their farm. One farmer stated, “*We get lessons from the Cocoa Extension Agents more frequently” “We now have easy access to pre-mix fuel to be able to spray our farm*”. COCOBOD has enabled the cooperatives to own and manage pruning machines and mist blowers, which the majority of the farmers could not afford to purchase individually. A cooperative member pays Ghc 10.00 towards repairs, which is approved by COCOBOD, to access and use these machines. Cooperatives are therefore effective means by which the adoption of practices can be encouraged by cocoa farmers, especially if these practices are supported by certification schemes, Licenced Buying Companies, or extension agents. Compared to widespread adoption of practices such as manual weeding, pruning, and intercropping, which are promoted by certification schemes, only 3% of cocoa farmers in Ghana and Côte d’Ivoire use organic fertilisers [56]. A possible reason for this low adoption of organic fertilisers may be because this practice is not supported by certification schemes, Licenced Buying Companies, or extension agents, and it is therefore not promoted by cooperatives. Cooperatives could therefore play an important role in dissemination of training in the production and use of organic soil amendments if this practice was supported by certification schemes, Licenced Buying Companies, or extension agents.

The majority (93%) of the respondents stated that they receive regular visits from the Cocoa Extension Agents in their operational areas (Table 4). The number of visits ranged from 2 to 4 times per week. The COCOBOD introduced an initiative that supports the pruning of the cocoa trees during 2017–2019 cropping seasons, since pruning is known to significantly increase cocoa productivity [5]. In all, 94.7% of the farmers were aware of this initiative and, amongst these, 98.8% of them were beneficiaries of the pruning exercise. The majority (96%) of the farmers indicated that they undertook pruning themselves when the need arose because of the benefit they have seen associated with the pruning of cocoa trees. This high adoption rate is similar to the rate (98.9%) observed by Buxton [57], but seems to contradict the findings of Obeng Adomaa et al., [58] who attribute the lack of adoption to the standardisation and de-contextualisation of pruning recommendations to conform with certification schemes promoted by commodity chains. Pruning by the farmers was encouraged and facilitated by the supply of machines for pruning to cocoa cooperatives. Farmers mentioned sunlight penetration and consequent reduction in disease incidence and increased yield as some of the benefits they have observed resulting from the pruning exercise. It is therefore plausible that the COCOBOD could follow the method described above to support the production and use of soil amendments by Ghanaian cocoa farmers, resulting in a self-sustaining practice if the farmers observe the benefits on their own farms.

On the use of pruning materials, 38% of the farmers indicated they do not use them for anything and leave them on the farm to rot to improve the soil fertility, while 62% of farmers indicated they do make use of the pruned materials as fuel wood for domestic purposes. During the focus groups several farmers indicated that they do not have any use for the pruned branches, which means that these materials are available for use as a biochar feedstock. It has been demonstrated that addition of biochar made from pruned cocoa branches can improve the growth of cocoa seedlings [59].

### Constraints to cocoa production

**Fig. 2:**
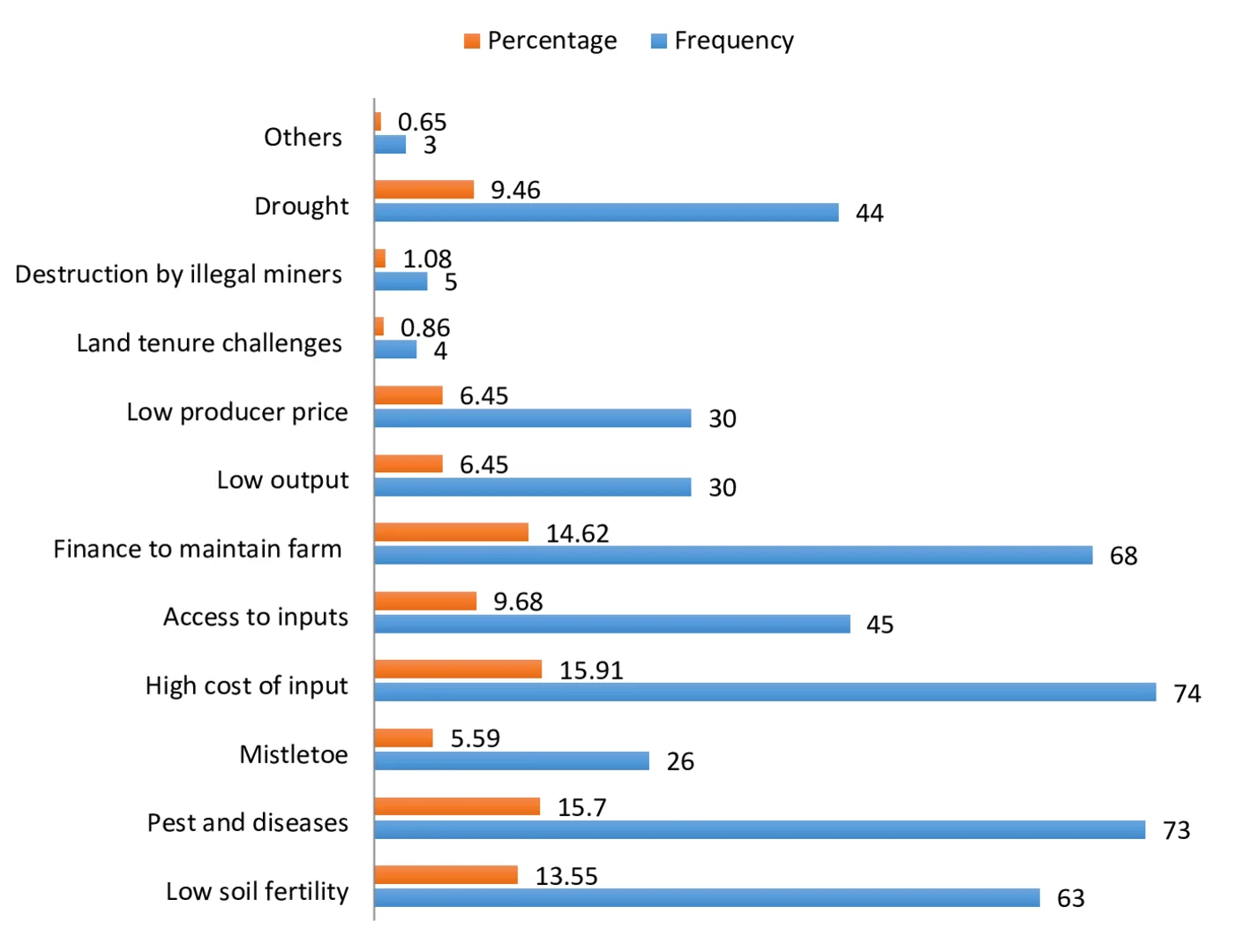
Constraints to cocoa production identified by questionnaire respondents

In response to multiple-choice questions, 15.9% of the respondents mentioned high cost of inputs and 15.5% mentioned pest and diseases as constraints to cocoa production in the study area (Fig. 2). These two factors were also identified by Kongor et al., [5] as the major constraints facing cocoa farmers in Ghana. Interestingly, 13.5% revealed low soil fertility as a constraint to cocoa production. Soil fertility decline was one of the major constraints that COCOBOD has acknowledged and this has led to the subsidisation of inorganic fertilisers by COCOBOD via registered distributers [26]. However, Kongor et al., [5] noted that continuous application of inorganic fertiliser has made the soil acidic. Consequently, there is a need to apply liming materials to alleviate the acidity. Snoeck et al., [25] noted that application of liming material would reduce soil acidity and improve nutrient availability to the plants. The application of biochar at appropriate rates to acidic tropical soils has been demonstrated to increase pH and enhance crop productivity, as an alternative to liming [60], although excessive applications need to be avoided to prevent the soil from becoming alkaline [61] because the optimal pH for cocoa is around 6.0 [25].

Given that the inputs used by the farmers are either for soil fertility improvement or the prevention of pests and diseases, it is not surprising that the highest percentage of the farmers reported high cost of inputs as one of the key constraints to cocoa production. About 14% of the respondents mentioned finance to maintain the farm as one of the major constraints. These results are similar to results obtained by Essougong et al., [46], which revealed that financial constraints, pest and diseases, poor soil fertility, and inability to access inputs are major constraints faced by cocoa farmers. Wilson et al., [62] similarly revealed that farmers find it difficult to access credit to purchase inputs, including fertiliser. Access to credit in particular is repeatedly observed to be a perceived barrier to technology adoption [27, 29, 63]. Cocoa farmer associations are a means by which farmers can access credit more easily [64]. However, despite regular applications of inorganic fertiliser, the farmers we interviewed still reported a decline in yield over time and so alternative, low cost approaches should also be considered to improve soil fertility.

All the farmers interviewed were aware that cocoa pod husks could be used to improve soil fertility. Our result confirms a study by Essougong et al., [46], which revealed that all cocoa farmers interviewed considered cocoa pod husks as a nutrient source for the cocoa trees. Indeed, returning the cocoa pod husks to the soil represents a considerable opportunity to close nutrient cycles and improve soil fertility [6, 65]. However, only 46% had made some attempt to use cocoa pod husks to improve soil fertility by leaving them on their farms. More than half (61.1%) of the respondents indicated that they leave the cocoa pod husks on the farm and do nothing to the pod husks, 28.6% of the farmers spread them on the farm, while 9.5% sell the pod husks or use them for soap making. In a focus group discussion, farmers revealed that they were worried about spreading diseases on the farm if they attempted to spread cocoa pod husks on the farm as an organic fertiliser to improve soil fertility, and this risk was reflected in guidance from the Cocoa Research Institute of Ghana which discouraged the use of cocoa pod husks as compost [66]. However, there is considerable evidence that composting cocoa pod husks supresses the Phytophthora pathogen and reduces the incidence of black pod disease [48, 67, 68]. Our survey showed that, despite this knowledge existing in the academic literature, none of the farmers make compost from cocoa pod husks. A greater understanding of the process of composting and preparation of biochar, and its ability to supress diseases, may eliminate farmers’ fear of using farm wastes as organic soil amendments. Since cocoa pod husks are low cost, internally generated, and are suitable for both composting and biochar production, their application to soils represents an opportunity to improve soil fertility at low cost while reducing nutrient leaching and greenhouse gas emissions [23].

### Awareness and use of organic soil amendments

Almost half (46%) of the farmers were aware that compost could be used as an organic fertiliser to improve soil fertility (Table 6). Most of the respondents (73.2%) indicated that they had heard of compost from the cocoa extension agents, 12.2% heard of compost from an NGO, while 5.7% of the farmers heard about it from other farmers (Table 5). In a focus group discussion, it was revealed that most of the farmers had heard of compost as a soil amendment for the production of vegetables, but not for application to cocoa trees. COCOBOD and NGOs established training for farmers in alternative livelihoods and it was during these training events that the cocoa extension agents and NGOs introduced them to the use of compost as an organic soil amendment in the production of vegetables, but not for cocoa. There are several studies that have demonstrated the benefits of cocoa pod husk derived composts for vegetable production, mostly in Nigeria [69–71]. However, it has also been demonstrated in Nigeria that the use of cocoa pod husk fertiliser can lower costs and increase revenue on cocoa farms [72]. The prior awareness of compost among Ghanaian farmers implies that existing extension services (i.e. cocoa extension agents and NGOs) could be used to disseminate information on the production and use of compost and biochar for cocoa trees to farmers.

With respect to the farmers’ knowledge on compost preparation, only 12.7% of those who were aware of compost as a soil amendment indicated they knew how to prepare it (Table 5). The focus group discussion revealed that, although they had heard of the method of compost preparation, they were not able to prepare it themselves for use on their farms without education and training. Ogunlade and Orisajo [68] integrated farmer training in the production of compost from cocoa pod husks as part of a participatory research experiment to demonstrate its beneficial impacts on yield and pathogen suppression. Therefore, participatory research methods may be an effective means by which the training of farmers in compost production may be achieved. Out of 81 farmers we surveyed who were not aware of compost, 96% of them were willing to learn how to prepare compost, while 4% were not willing because of the perceived cost associated with compost preparation. About 76% of the farmers surveyed were willing to buy compost as a soil amendment for use on their farms. This result highlights an opportunity for entrepreneurs who are interested in the business of compost preparation to meet a predicted increase in demand over the coming years [51]. Those who were willing to buy compost indicated, on average, that the price they were willing to pay for 25 kg compost was 26.33 Ghana Cedis. In a focus group discussion, the farmers suggested that COCOBOD should oversee production and distribution of the organic fertiliser via the cooperatives, as they do for other inputs including inorganic fertilisers and pesticides.

**Table 5:** Awareness and use of compost as organic soil amendment

Key issues investigated	Sub-level	Frequency	Percentage
Are you aware of compost?	Yes	69	46.0
	No	81	54.0
Which of these are your sources of information on compost (multiple response)?	COCOBOD CEAs	150	73.2
	LBCs	9	3.7
	NGOs	30	12.2
	Dealers in fertiliser	13	5.3
	Colleague farmers	14	5.7
	NGOs	1	14.3
	Others		
Do you have knowledge in compost preparation?	Yes	9	12.7
	No	60	87.3
Are you willing to learn how to prepare compost?	Yes	144	96.0
	No	6	4.0
Are you willing to purchase compost?	Yes	114	76.0
	No	36	24.0

Ghc – Ghana Cedis and 1 Ghc is equivalent to 0.0879 United States of America (USA) Dollars July 2023

**Fig. 3:**
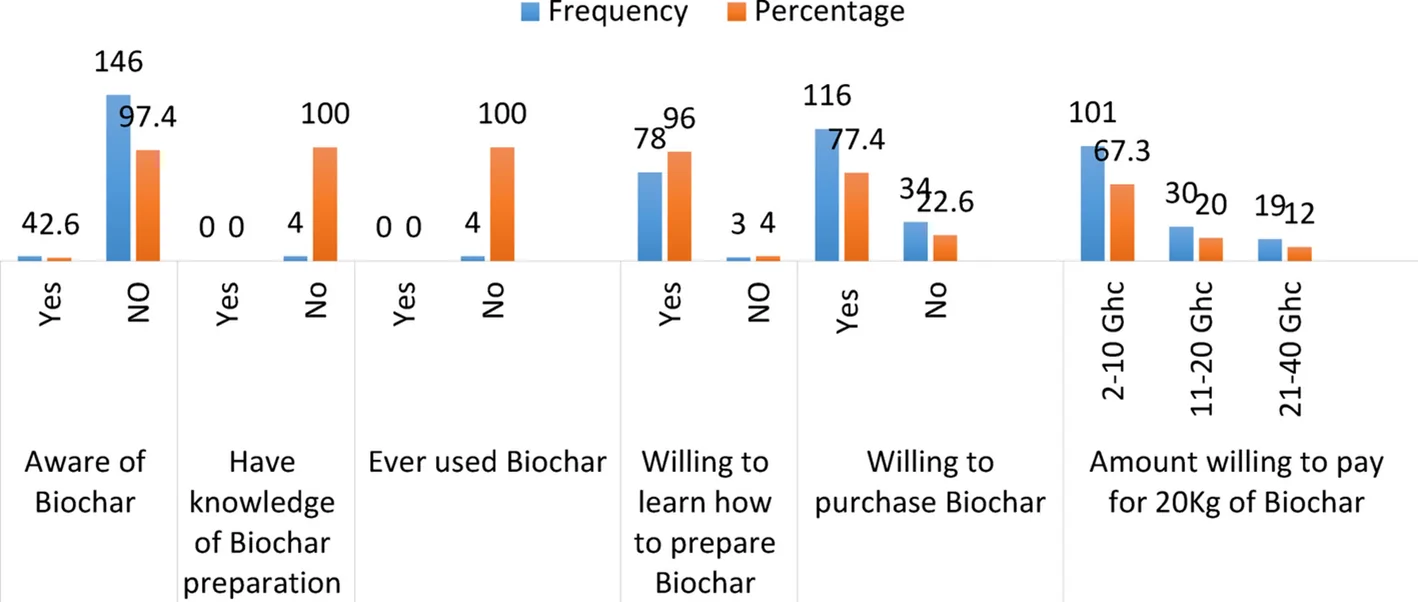
Awareness, use and wiliness to purchase biochar

The majority (97.4%) of the farmers surveyed had not heard of biochar as a soil amendment prior to the questionnaire (Fig. 3). Those who were aware of biochar indicated that they heard of it from an NGO who educated them on the use of organic soil amendments. None of the farmers interviewed had used biochar, nor had any knowledge of biochar preparation. Accordingly, introducing biochar to the farmers may be more difficult than the introduction of compost. As acknowledged by Yeboah et al., [73], the biochar supply and value chains are not formalised in Ghana and the product is not familiar to most farmers. However, 96.0% of the respondents were willing to learn how to prepare biochar while 77.4% were willing to buy biochar to improve soil fertility on their farms. About 67% of farmers willing to purchase biochar would pay 2–10 Ghana Cedis per 20 kg of biochar (Fig. 3). There is significant overseas interest in subsidising the cost of biochar production and use to offset greenhouse gas emissions elsewhere, but participation in these schemes may prioritise carbon markets over agronomic outcomes and may fuel ‘land grabs’ if the value of land for sequestering carbon using biochar becomes greater than the value to farmers for food production [74]. Furthermore, the use of biochar on some farms may not be appropriate since it has optimal benefits in soils which are acidic and infertile [60]. Adding biochar to soils with neutral pH can result in lower plant growth [61]. It is therefore recommended that soil testing is conducted prior to biochar application.

The farmers identified various constraints to the use of organic fertilisers including a lack of awareness (23.7%), unavailability of organic fertiliser compared with the inorganic fertiliser (19.6%), high expense (15.2%), lack of education (17.4%), and lack of labour (18.3%). A minority (6%) of the farmers perceived organic fertiliser as not being effective for improving soil fertility compared with inorganic fertiliser. As cocoa pod husks are readily available on farms and considered a waste product [75, 76], there is considerable opportunity for famers to use them for composting. However, there is a need to encourage the adoption of composting through education of the farmers about the benefits of using compost as a soil amendment in cocoa farms. Farmers were asked to rank the perceived constraints to the use of organic fertiliser (Table 6). The most important constraint ranked by the farmers was the bulky nature of the organic fertiliser, followed by labour for its application and the cost of the product, which included transportation of the organic fertiliser to the farms. It is likely that these constraints impact older farmers and those with less secure access to family labour (i.e. migrant or female farmers) disproportionately [77]. Research to identify optimal extension service delivery, business models, and logistical arrangements, should be prioritised to overcome these constraints.

**Table 6:** Perceived constraints to the use of organic soil amendments

Constraints	Mean score	Rank
Bulky	1.90	1st
Labour to apply	2.19	2nd
Too expensive	2.93	3rd
Aware but not available	4.48	4th
Lack of awareness	4.96	5th
Lack of education	5.64	6th
No impact on yield	5.91	7th
*Kendall’s W*	*0.588*	
*Asymptotic significance*	*0.000*	
*Df*	*6*	
*Chi square*	*543.002*	

### Limitations and generalisability of the study

This study was undertaken with a group of 150 farmers drawn from three selected operational areas and therefore caution should be applied before extrapolating the descriptive statistics reported here to all cocoa farmers in Ghana. However, the combination of the questionnaire and the focus group allows some specific deep insights to be drawn from the study which can inform the development of polices that raise awareness and adoption of soil amendments. Therefore, the insights arising from the study can be generalised. The farmers participating in the survey were already working with Cocoa Extension Agents from the Cocoa Health and Extension Division (CHED) of COCOBOD. However, not all cocoa farmers in Ghana have the same access to extension services. Only 25% of the participants in our study were female, and it is known that women farmers are less likely to have access to extension services [78]. Therefore, insights and interventions that are implemented as a result of this study should aim to meet the needs of marginalised communities with less access to information, training, and advice.

## Conclusion and recommendations

Whereas most farmers were aware of compost as an organic soil amendment for vegetable production, most were unaware of biochar and none of the 150 farmers surveyed had ever used compost or biochar on their cocoa farms. Most did not have the knowledge required to produce compost and none were able to produce biochar. Farmers indicated that they are willing to learn how to prepare compost and biochar after they were told it will improve productivity. Cocoa income contributes a large proportion of the farmers’ total household income and, although most farms were less than 15 years old, most farmers had noticed the productivity of their farms decline over time. We observed a high level of interest in innovations that will increase productivity. A programme of education and training for farmers on the preparation and application of compost and biochar as an organic fertiliser for improved cocoa productivity may increase the adoption of soil amendments by smallholder farmers. Once training has been provided the level of adoption of compost and biochar will likely be very high. However, several other barriers (e.g. the bulky nature, labour requirements, and transportation costs) were also raised by farmers. These barriers, combined with the knowledge that > 75% of farmers were willing to purchase biochar and compost but that there was currently nowhere to purchase it, highlights a considerable opportunity for the provision of subsidised soil amendments to play a key role in climate smart cocoa farming in Ghana.

Pre-existing structures and policies established by COCOBOD, cocoa extension agents, Licensed Buying Companies, and farmer associations (cooperatives) may be an effective vehicle to disseminate training to farmers on the preparation of compost and biochar. The establishment and management of demonstration farms where farmers can observe the effects of compost and biochar on performance of cocoa trees would likely encourage adoption and these demonstration farms could be established in collaboration between cocoa extension agents and cooperatives, with funding from Licensed Buying Companies. However, training programmes should be structured with an inclusive framework which acknowledges the social and environmental contexts within which individual cocoa farmers operate to prevent training disproportionately benefiting only well-resourced male farmers. Future research should investigate the most effective means to provide this training in an inclusive way.

The compost and biochar feedstock materials such as cocoa pod husks and pruned cocoa branches are available on farms and typically considered wastes. However, considering the bulky nature of organic amendments and the labour requirements to produce, transport, and apply these amendments on cocoa farms, there is a clear opportunity for entrepreneurs to establish businesses to produce, market, distribute, and apply compost and biochar as a service provided to farmers. Since the government, through COCOBOD, has historically provided services directly to cocoa farmers, they could support such enterprises with training and capital to establish businesses focused on the production and distribution of compost and biochar and through subsidisation of organic fertilisers for farmers. In line with the ‘One District One Factory’ policy of the Ghanaian government, a commitment to support at least one compost production plant in each district would encourage the production of compost for purchase by cocoa farmers. Licensed Cocoa Buyers and certification schemes may also include the use of compost and biochar within their requirements for cocoa farms that they work with and engage with the cooperatives to encourage the adoption of soil amendments by providing training and subsidising production through their farmer networks.

## Supplementary Information

Below is the link to the electronic supplementary material.


Supplementary Material 1


## Data Availability

The raw data associated with this manuscript cannot be made available to protect the identity of the participants.
