# Supplementary material for: Limited awareness prevents the use of compost and biochar as soil amendments by Ghanaian cocoa farmers
Source: Discov Agric. 2026 Aug 31;4(1):273. doi: 10.1007/s44279-026-00754-6 (PMC13529877; doi:10.1007/s44279-026-00754-6)
Supplement: Supplementary file 1 [file 44279_2026_754_MOESM1_ESM.pdf]

Supplementary material for:

Limited awareness prevents the use of compost and biochar as soil amendments by Ghanaian cocoa farmers

Dadson Awunyo-Vitor[1], Andrew Daymond[2], Amos Quaye[3], Godfred Kweku Awudzi[3], Laura Atuah[4], Lumbani Mwafulirwa[2], John Hammond[2], Chris Turnbull[2], Fiona Lahive[2], Sean Coole[5], Steve Robinson[5], Paul Hadley[2], Tom Sizmur[5*]

[1]Department of Agricultural Economics, Agribusiness and Extension, Kwame Nkrumah University of Science and Technology, Kumasi, Ghana

[2]School of Agriculture, Policy and Development, University of Reading, Reading, UK

[3]Cocoa Research Institute of Ghana, Tafo, Ghana

[4]Department of Horticulture, Kwame Nkrumah University of Science and Technology, Kumasi, Ghana

[5]Department of Geography and Environmental Science, University of Reading, Reading, UK

*Corresponding author (t.sizmur@reading.ac.uk) https://orcid.org/0000-0001-9835-7195

Content: This Supporting Information file contains:

- Participant questionnaire administered to 150 farmers
    - Page 2 to 20

- Questions asked in focus group with 8 purposively selected participants (2 male farmers with more than 10 years of cocoa farming experience, 2 female farmers with more than 10 years of cocoa farming experience, 2 farmers with at least high school level education, and 2 cocoa cooperative chair persons)
    - Page 21

- The method by which The Kendall's Coefficient of Concordance (W) was calculated
    - Page 22

GCRF- Cocoa Soil Project Questionnaire

Hello, my name is ……………………………………………………….. I am collecting data to understand your work as a cocoa farmer, farm size and production practices and yield levels. It is being conducted by a team from Kwame Nkrumah University of Science and Technology (KNUST) and Cocoa Research Institute of Ghana (CRIG) in collaboration with the University of Reading UK, with support from Global Challenge Research Fund (GCRF; a UK Government Research Fund). Your answers will be kept confidential.

A: IDENTIFICATION

A1 Name of Enumerator………………………………….

A2 Date of interview ……………………

A3 Record GPS of farm/community……………………..

B: DEMOGRAPHIC CHARACTERISTICS

B1. Farm location ……………………………………………

B2 Operational area ………………………

1. New Sanfo
2. Akropong
3. Sendu
4. Homaho
5. Kubekor
6. Assin Foso
7. Dadiesoaba
8. Mankesim
9. Acherensua
10. Pameng
11. Tweapease
12. Kade
13. Agona Namanwura
14. Kwanyako
15. Agona Mensakrom

B3. Cocoa District: 1. Aiyinase; 2. Assin Foso; 3. Dadiesoaba; 4. Kade; 5. Nyakrom

.

B4 Region: 1.Western South; 2. Central; 3. Bono; 4., Eastern

B5. Name of Respondent …………………………………………………….

B6 Telephone contact of respondent………………..

B7. Gender of the respondent

1=Male

2=Female

B8. Age of respondent in years …………………………………

B9 Is Respondent the Household Head?

1 = Yes

2=No

B10 For how many years have you lived in this community?................

B11 Are you a native of this locality?

1=Yes

2=No

B12 If you are not a native what is your residential status?

1=Migrant from Ghana

2= Migrant from outside Ghana

B13 Number of years in formal education……………………

B14 Highest level of Education attained

1=Never attended school

2=Primary school

3=Junior high school/MSLC

4=Senior high school/O' Level

5=Vocational Education

6=Tertiary (University, Polytechnic, etc.)

7=Koranic education

8=Other (specify)

B15. Marital status of the respondent:

1=Married

2=Consensual union/Co-habitation

3=Separated

4=Divorced

5=Widowed

6=Single/Never married

B16 Household composition (number of people living under respondents care)

Age Group & other measures	Gender		Total	Number not earning income	Number of adults
	Male	Female			
Under 15 yrs					
15-30 yrs					
> 30 <65yrs					
Above 65 years					
Total					
Dependency ratio	Dependency ratio: Number earning income/ number not earning income				
Adult Equivalent	= 1+0.7(N1) +0.5(N2) where N1= number above 15 years, N2= Number below 15 years				

B17 Do you belong to any farmer based organization

1=Yes

2= No

B18 if yes state the name of the organization ………………………….

B19 How many years have you been a member of this organization in …………..

B20 Do you have responsibility (serve on any committee) in any organization or within the society?

1=Yes

2= No

B21. If you have any responsible position kindly state the position …………………………………

1= chairman;

2= Vice chairman;

3= Treasure

4=secretary

5= member

6= other (specify)…………………………

B22 State the reason(s) for joining the organization ……………………………………………………

………

B23 Are you engaged in other income generation activity apart from cocoa farming?

1=Yes

2= No

B24 If you engage in other income (economic) activities please describe them? (select only one)

1= Self employed

2= employee

B25 if you are self employed which area

Sn#	Activities	Tick all as applicable	Rank from main activity 1 to minor activity 5		
1	Artisan				
2	Petty Trading				
3	By-day labourer				
4	Food processing				
5	Others				

C. COCOA PRODUCTION

C1 How many years have you been into cocoa cultivation?

C2 How many plots of Cocoa farm do you have?……………

C3 Complete the table below on cocoa cultivation

**Plot No**	**Plot size in Acres/Poles**	**Age of farm**	***Cocoa variety**	**Cocoa output in bags in past year**		****Nature of holding**	****If share cropping**
				Minor	Major		
1							
2							
3							
4							
5							

**Cocoa varieties: 1=Amelonado, 2= Hybrid (from SPU); 3= Mixture*

**Nature of holding: 1=inherited; 2= Lease, 3=Rent; 4=Share Cropping

*** Share cropping : 1=Abunu ; 2= Abusa)

C4 Which of these crops do you grow in addition to cocoa?

SN#	Crops	Tick as applied	Rank from larger proportion to smaller proportion 1-7	Remark
1	Plantain			
2	Cassava			
3	Cocoyam			
4	Yam			
5	Maize			
6	Vegetables			
7	Others (specify)			

C5 What percentage did cocoa contribute to total annual household income?...........................

C6 What percentage of available farmland used for cocoa cultivation?.........................

C7 What percentage of family labour used in Cocoa cultivation?..........................

C8 What percentage of total hired labour used in Cocoa cultivation?...................

C9 What percentage of total expenses that are related to Cocoa cultivation?...............

C10 Do you have contact with cocoa extension officers?

1=Yes

2=No

C11 If you do have contact with Cocoa Extension Agents (CEAs) how many times do you meet with the extension officer in the month? ………………………………………..

C12 How did you perceive soil fertility over the years

1= the same

2= declining

3= has improved

4=I can't tell

C13 Did you have any training on how to maintain soil fertility including use of fertilizer?

1 = Yes

2= No

C14 If yes, list the entity and the date of training (multiple response) tick as many as applied

**Sn #**	Training entities	**Thick as applicable**	**Order from major impact 1 to least impact 5**	**Remark**
1	Cocoa extension agents/ officers			
2	NGOs			
3	MoFA extension Agents			
4	Colleagues farmers			
5	Others –specify			

C15 if you think you have challenge with soil fertility what actions do you take to improve the soil fertility ……………………………………………………………………………………………..

………..

……….

C16 Indicate the soil amendment you use to improve soil fertility on your cocoa farm

1= inorganic fertilizer

2= Organic fertilizer

3= both inorganic and organic fertilizer

C17 What do you consider as organic fertilizer?...

…………………………….

……..

…….

C18 Have you ever experience major disease incidence on your farm recently

1 = Yes

2= No

C19 Did you use pesticides on your cocoa farm in the past twelve months?

1 = Yes

2= No

C20 If you use pesticide kindly indicate source of your pesticide supply

Sn#	Source of supply	Tick as applicable	Rank from 1 main supply source to 4 minor supply source
1	CODAPEC		
2	LBCs		
3	Co-operative own initiative		
4	Personal purchases		

C21 What are the constraints you faced in the use of pesticide and disease control?

Sn#	Source of supply	Tick as applicable	Rank from 1 major constraint to 7 minor constraint
1	Inadequate supply from CODAPEC- COCOBOD		
2	unavailability of supply from dealers		
3	Too expensive to purchase		
4	Lack of knowledge on the best pesticide to use		
5	Lack of equipment to apply the pesticide to control disease		
6	Ineffectiveness of the pesticide		
7	Lack of training on the use of pesticide		

C22 Are you aware of CCOBOD/ government tree pruning exercise

1 = Yes

2= No

C23 if you are aware has your trees being pruned?

1 = Yes

2= No

C24 If you are aware of the government pruning exercise but your tree has not been pruned kindly explain why?.. ....

……………………………………………………………………………………………………
…………………………………………….

C25 What is the effect of pruning on farm output

1=positive,
2= non change,
3=negative change

C26 Do you prune your cocoa trees yourself?

1 = Yes

2= No

C27 If you do prune your cocoa trees kindly indicate why you prune them……………………………

……………………………………………………………………………………………………

…………………………………………………………………………………………………..

C28 Do you make use of the pruned materials (either pruned by yourself or the government exercise)?

1 = Yes

2= No

C29 If you do make use of pruned material kindly state what you use the pruned materials for. …………………………………………………………………………………………………..

……………………………………………………………………………………………………

……………………………………………………………………………………………………

C30 If you do not prune cocoa trees on your farm kindly complete the table below

Sn #	Reasons	Thick as applicable	Order from 1-4 in order of Main reason to minor reason	Remark
1	Not aware of pruning			
2	Spread of disease			
3	Laborious work			
4	Do not know is good			
5	It will not have positive effect on yield			

C31 How did you use Cocoa Pod husk on your farm? Kindly complete the table below

**Sn #**	**Uses**	**Thick as applicable**	**Order from major use 1 to least use 7**	**Remark**
1	Spread on the farm			
2	Just heap on the farm			
3	Sell it			
4	ash it for soap making			
5	Do nothing to it			
6	Use it to make compost			
7	Others (specify)			

C32 Kindly complete the table on major/key constraints to cocoa production,

Sn#	Constraints	Tick as appropriate	Rank in order of Major constraint 1to least constrain 12	Remark
1	Declining soil fertility			
2	Pest and disease damage			
3	Mistletoe			
4	High cost of inputs			
5	Access to input on time			
6	Finance to maintain the farm			
7	Low output			
8	Low producer price			
9	Land tenure			
10	Destruction by illegal mining			
11	Drought			
12	Others, please specify:			

D: SOIL AMENDMENT (inorganic/organic fertilizer) USAGE BY FARMERS

D1 Can you generally identify poor soil (soil with declined fertility),

1= Yes

2= No

D2 If yes, which method did you use to identify poor soil ………………………………………

…………………………………………………………………………………………………….

……………………………………………………………………………………………………..

D3 if yes do you take any action to improve the fertility of the soil on your farm?

1 = Yes

2= No

D4 If you take action to improve the fertility of the soil, kindly explain the action to take.

…………………………………………………………………………………………………….

…………………………………………………………………………………………………….

…………………………………………………………………………………………………….

D5 What are the sources of chemical fertilizer for your cocoa farm?

Sn #	Sources	Tick as applicable	Rank from Main source 1to least 7
1	Cocobod from their office		
2	Cocobod via Cooperatives		
3	LBC via Cooperatives		
4	Cooperative initiative- purchases		
5	Supply by NGOs		
6	Dealers – Private purchase		
7	Others (specify)		

D6 What quantity of liquid inorganic fertilizer do you use per acre………………………

D7 What quantity of non-liquid inorganic fertilizer do you use per acre………………………

D8 if you do have training on the use of inorganic fertilizer who offers the training? Kindly complete the table below

Sn #	Training provider	Tick as applicable	Rank from the trainer with the highest impact 1to trainer with the least impact 7
1	Cocobod from their office		
2	Cocobod via Cooperatives		
3	LBC via Cooperatives		
4	Cooperative initiative- to organise training		
5	Training organised by NGOs		
6	Training organised by Dealers of organic fertilizer		
7	Others (specify)		

D9 What do you perceived as constraints in the use of chemical/ inorganic fertilizer. kindly complete the table below

	Reasons	Tick as appropriate	Rank the reasons in order of major reason 1 to minor reason 4	Remarks
1	Expensive			
2	Do not have access to it			
3	Not supplied by Cocobod at the right time			
4	Do not feel that it is important to use it			
5	Labour to apply it to the cocoa trees			
6	Others- specify			

D10 Are you aware of organic fertilizer?

1 = Yes

2= No

D11 If yes, which of these are you aware of (multiple response) tick as many as applicable?

1=Poultry manure.
2= Compost
3= Biochar
4= organic Fertilizer supplied by Cocobod
3= others (specify)....................

D12 Do you have any training on use of organic fertilizer?

1 = Yes

2= No

D13 if yes, indicate who offers the training by completing the table below

Sn #	Training provider	Tick as applicable	Rank from the trainer with the highest impact 1to trainer with the least impact 7
1	Cocobod from their office		
2	Cocobod via Cooperatives		
3	LBC via Cooperatives		
4	Cooperative initiative- to organise training		
5	Training organised by NGOs		
6	Training organised by Dealers of organic fertilizer		
7	Others (specify)		

D14 Have you ever applied or used organic fertilizer on your cocoa farm

1 = Yes

2= No

D15 What quantity of organic fertilizer do you use per acre (kindly state the unit of measurement ……………………………………………………………………………………………………

D16 if you use/ applied organic fertilizer to your cocoa farm kindly indicate the source by completing the table below

Sn #	Sources of organic fertilizer	Tick as applicable	Rank from Major source 1to minor source 8
1	Cocoabod/Cocoa Extension Agents		
2	Licenced Buying Companies (LBCs)		
3	NGOs		
4	Farmer Cooperative initiative		
5	Individual Purchased from Dealers		
6	Colleague Farmers		
7	Self prepared		
8	Others- specified		

D17 If you use organic fertilizer kindly state how you applied it to the cocoa trees

1= ring only
2=Broadcast
3= both Ring and Broadcast

D18 If you do not use organic fertilizer kindly explain why?………………………………

……………………………………………………………………………………………

……………………………………………………………………………………………..

……………………………………………………………………………………………..

……………………………………………………………………………………………..

D19 What do you perceived as constraints in use of organic fertilizer?

	Constraints	Tick as appropriate	Rank from major constraint 1 to the minor constraint 6	Remarks
1	Lack of awareness			
2	Aware but not available			
3	Do not feel it will have good impact on the yield			
4	Its too expensive			
5	Lack of education on it			
6	Labour to apply it to the cocoa trees			

D20 Do you know cocoa pod husk can be used to improve soil fertility?

1 = Yes

2= No

D21 If yes, do you use it?

1 = Yes

2= No

D22 if yes, kindly explain how the pod husk is used to improve soil fertility ……………………………………………………………………………………………

……………………………………………………………………………………………..

……………………………………………………………………………………………..

……………………………………………………………………………………………..

D23 If you know that cocoa pod husk can be used to improve soil fertility but you do not use it to improve soil fertility on the farm kindly complete the table below

	Reasons	Tick as appropriate	Rank from major reason 1 to minor reason 3	Remarks
1	Risk of spreading disease			
2	Insufficient labour to carry of the task of using the husk			
3	I have other uses for it			

E: COMPOST

E1 Have you ever heard of Compost?

1 = Yes

2= No

E2 If yes, indicate where (multiple response) tick as many as applied?

1=extension officers/ Cocobod
2= LBCs
3= NGO
4= Farmer Cooperative (they organize to invite expert to educate them)
5= from colleagues farmers
5= others (specify)…………………………..

E3 Can you prepare compost

1 = Yes

2= No

E4 If yes, explain the process

…………………………………………………………………………………………
……………………………………………………………………………………………..
………………………………………………………………………………………………
…..

E5 State materials you have used in compost preparation …………………………….

E6 If you ever used compost on your farm kindly state or indicate how you apply the compost to the Cocoa trees

1= ring only
2=Broadcast Only
3= both Ring and Broadcast

E7 If you do not know how to prepare Compost are you willing to learn how to prepare and use on your farm?

1 = Yes

2= No

E8 if you are willing to learn how to prepare Compost indicate the appropriate entity/ person to train farmers on how to prepare and apply compost

Sn #	Entity to offer training on compost preparation	Tick as applicable	Rank from most appropriate entity 1 to least appropriate entity 7
1	Cocoabod/Cocoa Extension Agents		
2	Licenced Buying Companies (LBCs)		
3	NGOs		
4	Farmer Cooperative to identify trainers		
5	Dealers in compost and fertilizer		
6	Colleague lead Farmers		
7	Others- specified		

E9 If you are ***NOT*** willing to learn how to prepare Compost, kindly explain why?............

…………………………………………………………………………………………………
………..………………………………………………………………………………………
……………………………………………………………………………………………..

E10 Are you willing to buy compost to apply to your cocoa trees

1 = Yes

2= No

E11 If you are ***NOT*** willing buy Compost, kindly explain why?...........

……..
………
……………………………………………………………………………………..

E12. If you are willing to buy compost how much are you willing to pay for a bag (fertilizer sack) of compost in Ghana Cedis ………………………….

F: BIOCHAR (*sample will be shown to the farmer by the enumerator*)

F1 Have you ever heard of Biochar?

1 = Yes

2= No

F2 If yes, indicates the source (multiple response)….……………………..

1=extension officers/ Cocobod
2= LBCs
3= NGO
4= Farmer Cooperative (they organize to invite expert to educate them)
5= from colleagues farmers
5= others (specify)…………………………..

F3 Can you prepare Biochar?

1 = Yes

2= No

F4 If yes, explain the process ……………………………………………

…………………………………………………………………………………………………..
………………………………………………………………………………………………….

F5 If you ever prepare Biochar what materials did you use …………………………………………

…………………………………………………………………………………………………..
…………………………………………………………………………………………………..

F6 If you ever used ***Biochar*** on your farm, kindly state or indicate the method used in applying Biochar to the Cocoa trees

1= ring only
2=Broadcast
3= both Ring and Broadcast

F7 If you do not know how to prepare Biochar are you willing to learn how to prepare and use on your farm?

1 = Yes

2= No

F8 if you are willing to learn how to prepare Biochar indicate the appropriate entity/ person to train farmers on how to prepare and apply compost

Sn #	Entity to offer training on Biochar preparation	Tick as applicable	Rank from most appropriate entity 1 to least appropriate entity 7
1	Cocoabod/Cocoa Extension Agents		
2	Licenced Buying Companies (LBCs)		
3	NGOs		
4	Farmer Cooperative to identify trainers		
5	Dealers in compost and fertilizer		
6	Colleague lead Farmers		
7	Others- specified		

F9 If you are ***NOT*** willing to learn how to prepare Biochar, kindly explain why?............

………..

………………………………………………………………………………………………………

……………………………………………………………………………………………..

F10 Are you willing to buy Biochar to apply to your cocoa trees

1 = Yes

2= No

F11 If you are ***NOT*** willing buy Biochar, kindly explain why?............

………..
……
……………………………………………………………………………………..

F12. If you are willing to buy Biochar how much are you willing to pay for five(5) Kilogram of a Biochar which is the same as (small bag of rice) in Ghana Cedis ………………………….

GCRF- Cocoa Soil Project Focus Group Questions

1. Explain why large number of immigrants are engaged in cocoa production

2. Any challenges you have faced as immigrants with respect to cocoa production

3. Why are some farmers not part of the co-operatives? And what can be done to ensure all farmers are part of the co-operative

4. What do farmers use the prune materials for?

5. What other uses for coco pod husks do the farmers have other than soap making and compost?

6. D you think training would persuade farmers to use cocoa pod husk for compost and biochar or you think there are other constraints that would still prevent them

7. You have more than 70% of your income from Cocoa but you have engaged in other income-generating activities, can you explain why?

8. What should be done to encourage use of organic fertilizer

9. Where and how did you hear of compost

10. Explain why farmers may be willing to learn how to prepare or buy compost and biochar?

11. How do you expect compost and biochar business models to operate so farmers can access compost and biochar

12. Will farmers be willing to make compost and biochar for sale.

13. What are the major constraints in use of compost and biochar

14. What should be done to improve the use of biochar and how can they be curtailed

The method by which The Kendall's Coefficient of Concordance (W) was calculated

Kendall's W ranges from 0 (no agreement) to 1 (complete agreement). In order to calculate W, the sum of ranks for each challenge being ranked is represented by T.

Based on the above, the variance sum of ranks can be specified as follows:

$$\mathrm{Var_T} = \frac{\sum T^2 - (\sum T)^2/n}{n} \quad \text{[Equation 1]}$$

where Var represents variance and n denotes the number of constraints or challenges being ranked by the farmers. The maximum variance of T can be specified as:

$$\frac{m^2(n^2-1)}{12} \quad \text{[Equation 2]}$$

where m represents the number of respondents. The formula for W (Kendall's coefficient of concordance) is given by:

$$W = \frac{\left(\sum T - (\sum T)^2/n\right)/n}{m^2 * (n^2-1)/12} \quad \text{[Equation 3]}$$

When equation 3 is simplified, it will result in the estimation formula for W as:

$$W = \frac{12[\sum T^2 - (\sum T)^2/n]}{mn^2(n^2-1)} \quad \text{[Equation 4]}$$
